# Voluntary Activation is Reduced in Both the More- and Less-Affected Upper Limbs after Unilateral Stroke

**DOI:** 10.3389/fneur.2014.00239

**Published:** 2014-11-19

**Authors:** Jocelyn L. Bowden, Janet L. Taylor, Penelope A. McNulty

**Affiliations:** ^1^Neuroscience Research Australia, Sydney, NSW, Australia; ^2^University of New South Wales, Sydney, NSW, Australia

**Keywords:** maximal voluntary contraction, biceps brachii, neural drive, motor cortex, motor evoked potential

## Abstract

**Objective:** Measurement of voluntary activation gives an indication of neural drive to the muscle. This study aimed to identify the site of impairment in neural drive during voluntary contractions post-stroke.

**Methods:** Elbow-flexor voluntary activation was assessed bilaterally for 10 stroke patients (mean 61.2 ± 12.3 years) and 6 age-matched controls (61.3 ± 14.0 years) by stimulating either the peripheral nerve or the motor cortex during maximal voluntary contractions. Any additional evoked force during maximal contractions implies neural drive is incomplete. Peripheral stimulation can detect deficits at or above the stimulation level, while cortical stimulation can identify suboptimal supraspinal output.

**Results:** Impairments were apparent on the less-affected side in addition to the more-affected side after stroke in voluntary activation, torque, and electromyographic activity (EMG) response. Maximal torque was reduced by 44% on the more-affected and 31% on the less-affected side compared to healthy controls (*p* < 0.001). Peripheral voluntary activation was reduced to 81% on the more-affected side and 86% on the less-affected side, with healthy subjects at 96% (*p* < 0.05). Although EMG was bilaterally impaired after stroke, the pattern of response was different between sides. Voluntary activation could not be calculated for cortical stimulation post-stroke due to variability in the evoked force, but EMG results from cortical stimulation showed significant differences in the neural drive to each side.

**Conclusion:** Voluntary activation is impaired bilaterally in the upper-limb after stroke, with reduced cortical connectivity on the more-affected side.

**Significance:** Although the muscle itself did not change post-stroke, altered descending drive and connectivity were the critical factors explaining post-stroke paresis.

## Introduction

Voluntary movement is contingent on the ability to generate and control muscle force. To optimize motor performance commands from the cortex must be transmitted with high fidelity to the peripheral neuromusculature via the descending tracts. Damage to any component of this pathway from stroke can impair voluntary movement (1). Hemiparesis, or muscle weakness on one side of the body, is the most common outcome after stroke (2–4), although deficits can also occur on the ipsilesional or less-affected side (5–9). The exact mechanisms of post-stroke weakness are not fully understood, although reduced neural drive to the muscle may be a significant contributor. Changes in neural drive may arise from a decrease in cortical output as a result of the stroke lesion (10, 11); a reduction in the connectivity of the descending pathway (12); or secondary long-term adaptive changes in the peripheral muscles, nerves, and joints (13). Although these changes are known to occur, the exact contribution of supraspinal and peripheral impairments to post-stroke weakness are not known, primarily due to the difficulty of measuring cortical output in humans. Voluntary activation is a measure of the neural command to a muscle during a voluntary effort and is one of the few non-invasive tools available that can identify impairments in neural drive.

Voluntary activation can be assessed using two different sites of stimulation: peripheral or cortical. The traditional peripheral method applies a percutaneous electrical stimulus to the motor nerve of the muscle during a maximal voluntary contraction (MVC) (14, 15). If the peripheral stimulus evokes an additional torque, known as a superimposed twitch, either recruitment of the motoneurones is incomplete or they are firing at less than maximal tetanic rates (16–18). Yet peripheral nerve stimulation cannot identify the site or mechanism of impaired voluntary drive (19). Therefore, a newer voluntary activation technique that uses transcranial magnetic stimulation (TMS) of the motor cortex has been used in healthy subjects (19, 20). This method can also assess the connectivity in the descending corticospinal tracts, and the ability of the central nervous system to drive those connections during a voluntary contraction. An increase in the size of the superimposed twitch evoked with the cortical stimulation suggests suboptimal supraspinal output whereas a decrease in the amplitude of the motor evoked potentials (MEPs) measured in the muscle may indicate impaired anatomy or functional connectivity of the descending tracts (19, 20). Functional connectivity is defined as the temporal correlation between spatially separated neurophysiological events (21).

Peripheral nerve stimulation after stroke has demonstrated reduced voluntary activation on the more-affected side of 48–86% for the lower limb (22–26) while 66% was reported in a single upper-limb study (27). Despite evidence of bilateral upper-limb weakness after stroke, the less-affected side has only been studied twice, both in the lower limb, with voluntary activation reduced to ~75% (22, 23). Although the majority of strokes occur after 60 years of age (28, 29) these reductions are not thought to be age-related, as voluntary activation in older healthy subjects is reported at 90–96% (30–35). However, this hypothesis has not yet been directly addressed. The only two previous studies that compared voluntary activation in stroke patients to healthy age-matched subjects used peripheral stimulation and were undertaken in the lower limb (22, 23). In contrast, cortical stimulation has only been used previously to assess voluntary activation in healthy subjects (19, 34, 35), it has not been used after stroke. This is surprising given TMS studies are commonly used to investigate the functional integrity of the corticospinal pathway and excitability of corticospinal projections post-stroke (1, 12, 36, 37). The use of TMS also has the advantage of allowing the muscle properties that may contribute to reduced voluntary activation to be investigated during the MVC. Electromyographic activity (EMG) responses to peripheral nerve stimulation have not been well characterized in voluntary activation studies as stimulation over the muscle typically precludes the measurement of EMG [but see in Ref. (23)].

The aim of this study was to investigate the contribution of peripheral and supraspinal mechanisms to post-stroke muscle weakness in the elbow flexors using voluntary activation techniques. This is the first study to assess the level of voluntary activation in both elbow flexors of stroke patients and healthy age-appropriate control subjects, using both peripheral nerve and cortical stimulation. The contractile properties and EMG characteristics of the stimulated muscles were also investigated. We hypothesized that patients would have reduced cortical voluntary activation on both sides and that this would reflect changes in descending cortical drive and the consequent EMG response.

## Materials and Methods

The elbow-flexor muscles of 10 stroke patients were studied (aged 39–75 years, 4–123 months post-stroke, see Table 1) using both peripheral and cortical stimulation. Patients had hemiparesis that included an upper-limb impairment following a unilateral stroke in the territory of the middle cerebral artery. Six patients were on statin medications. Another patient was assessed but these data were not included in the analysis as the responses to cortical stimulation were the opposite of those found in both healthy subjects and other stroke patients (i.e., the amplitude of the superimposed twitch *increased* with greater torque, rather than decreased). This finding was consistent when repeatedly tested over a 6-month period. The results for stroke patients were compared to bilateral assessments in six neurologically healthy, age- and sex-appropriate control subjects aged 42–73 years, including one of the authors (Table 1). Cognitive competency for all participants was assessed as a score ≥24 on the Mini-Mental State Examination. All participants gave written, informed consent. Ethical approval was given by the Human Research Ethics Committees of St. Vincent’s and Prince of Wales Hospitals, Sydney, Australia. Informed consent was obtained from all participants and all experiments were conducted in accordance with the Declaration of Helsinki.

**Table 1:** **Demographic data and functional scores of patients and healthy subjects**.

	Stroke patients	Healthy subjects
*n*	10	6
Age	61.2 ± 12.3	61.3 ± 14.0
Sex (male:female)	8:2	4:2
Months post-stroke	17.6 ± 17.4	n/a
Dominant side (right:left)	8:2	6:0
More-affected side (right:left)	5:5	n/a
Dominant side affected	7	n/a
Stroke type ischemic:hemorrhagic	7:3	n/a
Wolf Motor Function Test (s)	11.2 ± 20.0	n/a
Fugl-Meyer assessment	56.5 ± 5.3	n/a
MALQOM	89.2 ± 42.4	n/a
Modified Ashworth scale
Elbow (*n* = 7 > 0)	1.5 [0–2.0]	n/a
Shoulder (*n* = 8 > 0)	1.0 [1–1.5]	
Arm circumference (mm) (ma:la, dom:nd)	293:301	292:281

*Age, months post-stroke, and functional data are reported as mean ± SD. The modified Ashworth scale as median (interquartile range). Functional data were collected for the more-affected side only. Wolf Motor Function Test data are the mean of the summed times for the 15 timed tasks; maximum Fugl-Meyer score is 66; maximal MALQOM score is 150. Modified Ashworth scores are presented for the elbow and the shoulder on a six-point scale where 0 = no spasticity and 4 = affected part(s) rigid in flexion or extension. ma, more-affected side; la, less-affected side; dom and nd, dominant and non-dominant hands, respectively of healthy subjects*.

### Experimental procedures

**Figure 1:**
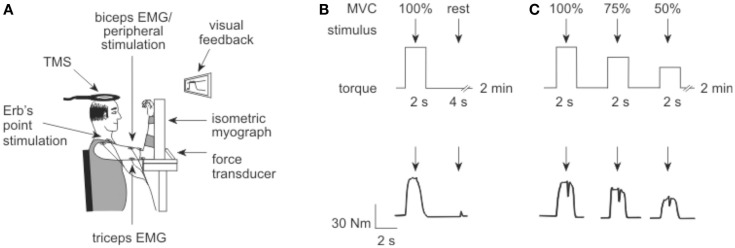
**Experiment set up**. **(A)** Elbow flexion torque was recorded with an isometric myograph. Electrical stimulation was delivered to Erb’s point and the motor nerve of biceps, while transcranial magnetic stimulation (TMS) was delivered over the motor cortex. **(B)** To measure voluntary activation in the first protocol, electrical stimuli (arrows) were delivered to the motor point of biceps brachii during and after a brief MVC. The protocol is illustrated above, with a torque trace from a participant shown below. **(C)** To measure voluntary activation in the second protocol, TMS (arrows) was delivered over motor cortex during a 100, 75, and 50% MVC, approximately 4–5 s apart. Again, the protocol is illustrated above with a participant’s torque record shown below.

The experimental protocols used in this study were designed to replicate the work of Todd and colleagues in healthy subjects (19, 20). Participants sat with their forearm securely strapped to an isometric myograph (Figure 1A). The elbow and shoulder were flexed to ~90° with the forearm vertical and supinated. If patients were unable to achieve this due to stiffness they were positioned as closely as possible without discomfort. Slightly more acute joint angles of 80–90° occurred on the more-affected arm of two patients. Bilateral assessments were performed during the same 2.5 h session.

#### Torque and EMG recordings

Elbow flexion torque (Newton meter) was measured using a 2 kN isometric load cell (Xtran, Applied Measurements Australia), low-pass filtered at 20 Hz, amplified 550–1760 times and sampled at 2000 Hz. EMG was recorded with surface electrodes from the biceps brachii and triceps brachii in a muscle belly tendon arrangement. Surface EMG signals were amplified 200–300 times, filtered from 10 to 1000 Hz using a 1902 amplifier (CED, UK) or IP511 amplifier (Grass, USA) and sampled at 5000 Hz. Torque and EMG were recorded and digitized using a 1401 digital analog converter and Spike2 software (CED, UK).

### Experiment protocol

After familiarization with the experiment protocols, tests were completed in the order described below. The less-affected arm of stroke patients or the dominant arm of healthy subjects was tested first to gain familiarity with the protocol on the better performing side. Maximal elbow-flexor torque for each side was determined as the peak torque produced during two to three brief (2–3 s) MVCs performed with strong verbal encouragement and visual feedback. The torque target for contractions at 100, 75, and 50% MVC was displayed on-screen for subsequent trials. Care was taken to ensure the MVC amplitude occupied ~1/3 of the screen regardless of the absolute torque. Participants were instructed to pull as hard as they could for the MVC, but to match the target display for the 75 and 50% efforts.

Three forms of stimulation were used throughout the study: peripheral electrical stimulation at the brachial plexus and at the motor point of the biceps; and stimulation of the motor cortex using TMS. The stimulation protocols are illustrated in Figures 1B,C.

#### Peripheral nerve stimulation of the brachial plexus at Erb’s point

During the experimental setup single pulse electrical stimuli, 0.1 ms pulse width (DS7AH, Digitimer, UK), were delivered to Erb’s point via a cathode over the brachial plexus in the supraclavicular fossa. The anode was placed over the acromion. A stimulus–response curve was undertaken to determine the maximal compound muscle action potential (M_max_) of the resting biceps and triceps muscles. The electrical stimulus was increased from 2.5 to 5 mA and thereafter in 10 mA increments until M_max_ was reached. The amplitude of M_max_ was required to normalize peripheral and cortically evoked EMG measures (see Data Analysis).

#### Peripheral nerve stimulation of the biceps

During the peripheral stimulation protocol single pulse supramaximal electrical stimuli, 0.1 ms pulse width (DS7AH, Digitimer, UK), were delivered to the intramuscular branches of the musculocutaneous nerve innervating the biceps. The stimulating electrode for the biceps was located at approximately two–thirds of the distance between the axillary and elbow creases in the midline of the biceps muscle, with the anode on the biceps tendon. A stimulus–response curve was performed as described above to define the current required to produce a resting twitch of maximal amplitude in the unpotentiated biceps muscle. The supramaximal stimulation intensity was set to 120% of the level required to produce the maximal twitch. Once set for each side, stimulator intensity remained at this level for the duration of the experiment.

#### Voluntary activation measured with peripheral nerve stimulation

Peripheral voluntary activation was assessed from five MVCs performed with a 2-min rest between contractions to avoid fatigue (Figure 1B). A single supramaximal electrical stimulus was delivered to the motor point of the biceps muscle during the plateau of the MVC torque, followed by a second stimulus delivered 4 s later to the relaxed muscle.

#### Transcranial magnetic stimulation of the motor cortex

Transcranial magnetic stimulation was applied to the motor cortex (Magstim 200^2^, Magstim Co, UK) to elicit MEPs in the biceps and triceps. A circular coil of 12.5 cm external diameter was positioned and held by the experimenter over the vertex (Figure 1A) with the direction of current flow set to preferentially activate the motor cortex supplying the side to be tested. Firstly, the site over the cortex that produced the greatest MEP response was located. This was generally achieved with the muscle at rest. If a MEP could not be evoked at rest, a weak voluntary effort ~5% MVC was used to facilitate the response. Once a suitable site was located all cortical stimulation was delivered during voluntary contractions according to the protocols described below. To minimize any antagonist twitch, the site of stimulation and the level of stimulator output were optimized to produce a MEP in the biceps of >50–60% M_max_ during a 50% MVC, and a MEP in the triceps of <10–15% M_max_ during an MVC of the elbow flexors. These target MEPs were not always achievable in patients, so the location and stimulus intensity producing the largest difference between the biceps and triceps MEPs was selected. After the optimal site was established, the coil position was marked on the head allowing accurate repositioning for each stimulus. A stimulus–response curve was performed to determine the optimal stimulator output for measurement of voluntary activation. Participants were instructed to perform a series of brief contractions of ~50% MVC. During each contraction a single magnetic stimulus of increasing intensity was delivered during a plateau in the force. Stimulus intensity commenced at 30 with 5% increments until 95% of stimulator output was reached. Once the stimulus site and intensity were determined separately for each arm, they remained constant for the duration of the experiment.

#### Voluntary activation measured with cortical stimulation

As cortical and motoneuronal excitability increases during a voluntary effort, the amplitude of the cortically evoked resting twitch must be estimated rather than measured directly (19, 20). The resting twitch was estimated from a linear regression between the amplitude of the superimposed twitch (Newton meter) and the voluntary torque (Newton meter) evoked at 100, 75, and 50% MVC. Contractions were performed at each intensity with ~5 s between contractions, and 2 min rest between each of the five sets. Each set contained contractions performed in order from 100 to 50% MVC to ensure maximal muscle potentiation. A single cortical stimulus was delivered over the motor cortex during a plateau in the torque of each contraction.

#### Functional assessments for stroke patients

The functional ability of stroke patients was assessed on the more-affected side on a separate day prior to voluntary activation testing (Table 1). Functional assessments included the Wolf Motor Function Test (WMFT) (38) and the upper-limb motor subscale of the Fugl-Meyer Assessment (39). Muscle resistance at the elbow and shoulder were assessed on the more-affected side using the modified Ashworth scale (40). To provide some indication of muscle atrophy, the circumference of each upper arm was measured at the point of greatest girth while the arm was strapped into the myograph. Use of the more-affected side was quantified using the Motor Activity Log Quality of Movement scale (MALQOM), a self-rated questionnaire consisting of 30 activities of daily living (41). It is scored on a six-point scale with 0 representing an inability to complete the task and five representing the same ability as pre-stroke.

### Data and statistical analysis

#### Torque

Torque signals were smoothed using a 10-ms time constant. Maximal voluntary torque was measured as the peak amplitude prior to the stimulus for 100% contractions, and over 50 ms prior to the stimulus for 75 and 50% contractions. The amplitude of the superimposed and resting twitches was calculated as the difference between the twitch peak and the mean torque for 50 ms immediately prior to the stimulus for all contraction strengths. To allow inter-subject comparisons twitch amplitude was normalized to the amplitude of the largest MVC recorded for that side (% MVC). The time-to-peak of the superimposed twitch and resting twitch were measured (milliseconds) with the onset taken from the stimulus to provide an unambiguous measurement point. This was particularly important when measuring time-to-peak of the superimposed twitch evoked with peripheral nerve stimulation as the exact twitch onset was not always evident during high activation. Half-relaxation time of the resting twitch was measured from the peak of the twitch to the point where the twitch torque was reduced by 50%. The estimated resting twitch was calculated from a linear regression between the amplitude of the superimposed twitch (Newton meter) and the voluntary torque (Newton meter) at 100, 75, and 50% MVC. The *y*-intercept of the regression equation was taken as the amplitude of the resting twitch (19, 20). To provide a quantitative measure of twitch amplitude variability, the mean standard deviation for the five twitches evoked with both peripheral and cortical stimulation at 100% MVC was calculated for each subject and for each side.

#### Voluntary activation

The level of voluntary activation was calculated for both peripheral and cortical stimulation methods using the interpolated twitch technique: voluntary activation (%) = (1 − superimposed twitch/(estimated) resting twitch) × 100.

Electromyographic activity and MEPs were recorded from the biceps and triceps during cortical stimulation and analyzed for participants who completed bilateral assessments. EMG could not be recorded during peripheral voluntary activation due to stimulation at the biceps motor point. The DC offset was removed from the EMG signal before root mean square (RMS) processing using a 10-ms time constant. The mean of the background noise in the EMG signal was measured over 500 ms and subtracted from EMG values. RMS EMG was measured as the mean amplitude over 500 ms immediately prior to the stimulus, and normalized to the amplitude of M_max_ (% M_max_). The area of M_max_ and the MEPs were measured between set cursors for both the biceps and triceps muscles. The ratios of the triceps to biceps EMG and MEPs were calculated to gauge co-contraction of the agonist and antagonist muscles. Finally, the duration of the silent period following cortical stimulation was measured in the biceps EMG from the stimulus to the return of continuous EMG. MEP responses evoked with cortical stimulation were normalized to M_max_ to enable between subject comparisons.

Data were analyzed using separate two-way ANOVAs for torque, EMG, and MEP data. Factors for the torque data were side-tested and stimulation type. Factors for the EMG and MEP data were side-tested and contraction level (% MVC). Results are presented as mean ± standard error of the mean (SEM) with Holm–Sidak *post hoc* analyses. As there were no significant differences between the dominant and non-dominant sides of healthy control subjects for any measure, the data have been combined and the pooled results are referred to throughout. Results were considered significant when *p* < 0.05.

## Results

### Functional assessments

Functional assessments scores for the patient group are reported in Table 1. The WMFT and the Fugl-Meyer Assessment scores indicate high level post-stroke functional ability (42, 43). Eight patients had increased muscle resistance at the more-affected elbow and/or shoulder. There was no significant difference in the upper-arm circumference between sides.

### Maximal voluntary torque

**Figure 2:**
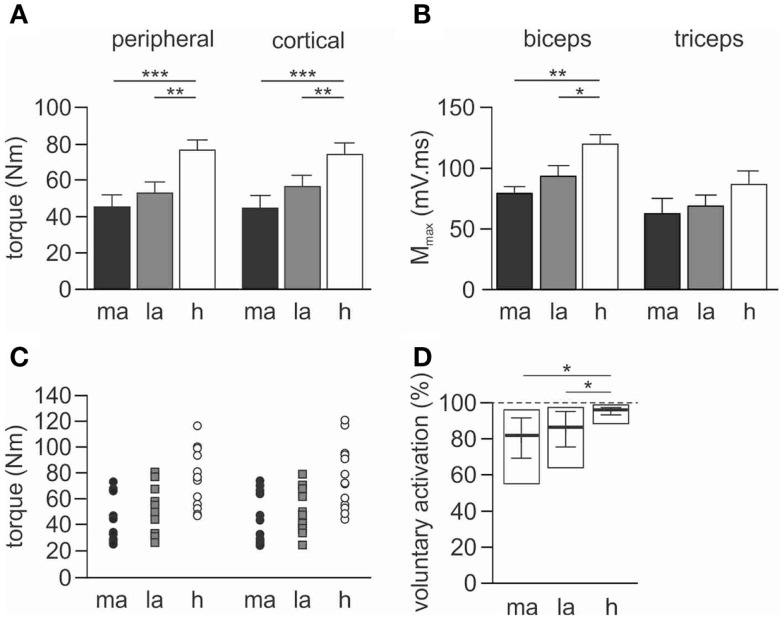
**Maximal torque, M_max_ and voluntary activation during peripheral nerve stimulation**. Data for torque and M_max_ are presented as mean ± SEM, and voluntary activation as median and interquartile range. **(A)** Maximal voluntary torque (Newton meter) generated during the 100% MVC for both peripheral and cortical stimulation protocols. **(B)** Maximal compound muscle action potential (M_max_) evoked in the resting muscles during stimulation at Erb’s point. Results are shown for the biceps and triceps muscles. **(C)** Individual maximal voluntary torque data for both peripheral (circles) and cortical (squares) stimulation protocols. **(D)** Pooled voluntary activation scores calculated from peripheral nerve stimulation. The open rectangle represents the maximum and minimum scores for each group calculated from the mean of the five trials. The solid horizontal line is the median score with the interquartile range. The dashed line represents the highest possible score at 100% voluntary activation. ma, More-affected side, la, less-affected side, h, healthy subjects. *p < 0.05, **p < 0.01, ***p < 0.001.

Torque demonstrated an effect for the side-tested (*p* < 0.001, Figures 2A,C). Maximal torque was reduced on both the more-affected (*p* < 0.001) and less-affected sides (*p* = 0.003) of stroke patients compared to healthy subjects, but there was no statistically significant difference between the more- and less-affected sides. There was no effect for protocol (peripheral or cortical stimulation) and no interaction with side-tested. The similarity in the maximal torque produced during the peripheral and cortical protocols for both groups suggests fatigue did not influence the results (Figure 2A). Importantly for the cortical protocol all participants could appropriately grade torque output to 75 and 50% MVC.

### EMG maximal muscle response to brachial plexus stimulation

There was an effect of side-tested for biceps M_max_ area (*p* = 0.002) but not for triceps M_max_ area. Biceps M_max_ was smaller for both the more-affected (*p* = 0.002) and less-affected biceps (*p* = 0.040) when compared to healthy subjects, but this was not different between more- and less-affected sides in stroke patients (Figure 2B).

### Peripheral nerve stimulation

#### Stimulus intensity

The current required to evoke M_max_ during Erb’s point stimulation and biceps motor point stimulation was not significantly different either within or between groups. The mean current at Erb’s point was 46.0 ± 6.7 mA on the more-affected side, 51.0 ± 8.2 mA on the less-affected side, and 56.7 ± 7.5 mA for healthy subjects. The mean current at the biceps motor point was 64.0 ± 4.9 mA on the more-affected side, compared to 62.0 ± 6.3 mA on the less-affected side, and 57.5 ± 4.9 mA for healthy subjects.

#### Resting twitch

**Figure 3:**
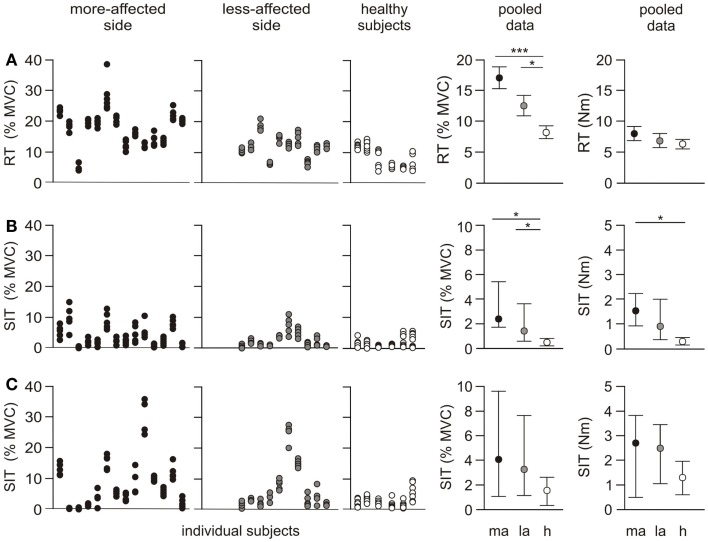
**Individual and pooled data for the resting twitch and superimposed twitch**. The first three panels in each row represent the amplitude for each twitch for individual subjects. Five trials were undertaken for each protocol on both sides, and are normalized to the highest MVC for the side-tested (% MVC). The fourth and fifth panels show the pooled results as a % MVC, and torque (Newton meter). The pooled resting twitch data (% MVC) are presented as mean ± SEM. Resting twitch (Newton meter) and both rows of superimposed twitch data are presented as median and interquartile range. **(A)** Resting twitch (RT) evoked with peripheral nerve stimulation over biceps brachii **(B)** superimposed twitch (SIT) evoked with peripheral nerve stimulation over biceps brachii **(C)** superimposed twitch evoked with cortical stimulation. *p < 0.05, ***p < 0.001.

The resting twitch was not significantly different either between or within groups for amplitude (Newton meter), time-to-peak, or half-relaxation time (Figure 3A; Table 2). When expressed relative to MVC the amplitude of the resting twitch demonstrated an effect for side-tested (*p* < 0.001). Twitches on both the more- (*p* < 0.001) and less-affected sides (*p* = 0.038) were larger than in healthy subjects, and were also different to each other (*p* = 0.041). There was a side-tested effect for the mean standard deviation of the resting twitch amplitude (*p* < 0.001). Both the more-affected and less-affected sides were more variable when compared to healthy subjects (*p* < 0.05, Table 2), but not different to each other.

**Table 2:** **Summary of the resting twitch and superimposed twitch characteristics, MEP onset latency and silent period duration**.

	Stroke patients		Healthy subjects
	More-affected	Less-affected	Pooled data
	side	side	
**Peripheral stimulation**
RT time-to-peak (ms)	96 ± 3	95 ± 5	97 ± 2
RT_HRT_ (ms)	83 ± 7	71 ± 8	75 ± 5
RT_SD_	1.2 (0.7–1.8)*	0.9 (0.7–1.2)*	0.4 (0.2–0.5)
SIT time-to-peak (ms)	67 ± 3**	61 ± 7	53 ± 3
SIT_SD_	1.7 ± 3*	0.9 ± 3	0.5 ± 1
**Cortical stimulation**
SIT time-to-peak (ms)	91 (84–92)*	77 (62–91)	76 (44–83)
SIT_SD_	2.1 (1.1–3.7)*	1.2 (1.0–2.1)**	0.6 (0.4–1.0)
**MEP onset latency (ms)**
Biceps	12.1 ± 0.3*	10.9 ± 0.3	10.9 ± 0.3
Triceps	14.2 ± 0.5*	13.6 ± 0.5*	12.6 ± 0.5
Silent period biceps (ms)	250 ± 22	170 ± 22*	220 ± 20

Time-to-peak results are measured from the stimulus. Data are reported as mean ± SEM or median and interquartile range. RT, resting twitch; SIT, superimposed twitch; _HRT_, half-relaxation time; _SD_, mean of the standard deviation of the amplitude of five twitches for each subject. The MEP onset latency and biceps silent period were measured during the 100% MVCs. *p < *0.05* compared to healthy subjects, **p < *0.08* compared to healthy subjects.

#### Superimposed twitch

There was an effect for side-tested for the amplitude of the superimposed twitch (*p* = 0.004). The amplitude was larger on the more-affected side compared to healthy subjects (*p* < 0.05), but not compared to the less-affected side (Figure 3B). The effect for side-tested was greater when the superimposed twitch amplitude was normalized to the MVC amplitude (*p* < 0.001). The twitch on both the more- and less-affected side was larger than that of healthy subjects (*p* < 0.05), but the sides were not different to each other. There was a significant effect for side-tested for the within-subject standard deviations of the superimposed twitch amplitude (*p* = 0.006), with variability higher on the more-affected side compared to healthy subjects (*p* < 0.05, Figure 3B; Table 2) but not between sides in the patient group. There was a trend for the time-to-peak of the superimposed twitch (*p* = 0.073, Table 2) to be slower for the more-affected side compared to healthy subjects.

#### Voluntary activation measured with peripheral nerve stimulation

Overall there was an effect for side-tested (*p* = 0.003). Voluntary activation scores were lower on both the more- and less-affected sides compared to healthy subjects (*p* < 0.05, Figure 2D), but there was no difference between sides for stroke patients.

### Cortical stimulation

#### Pre-stimulus EMG

Biceps EMG demonstrated an effect of side-tested (*p* < 0.001), but triceps EMG did not (Figure 5A). Biceps EMG was smaller for the more-affected side than both healthy subjects and the less-affected side (*p* < 0.05). There was no difference between the less-affected side and healthy subjects. There were no interactions with contraction level.

The ratio of triceps to biceps EMG demonstrated an effect for side-tested (*p* = 0.008) being larger on the more-affected side compared to healthy subjects (*p* = 0.006). There was no difference between the less-affected side and healthy subjects and no interaction with contraction level. During maximal contractions, the ratio on the more-affected side was 43.2 ± 8.4%, compared to 33.3 ± 8.4% on the less-affected side and 26.0 ± 7.3% in healthy subjects. The larger EMG co-contraction ratios in the patient group were due to decreased activity of the biceps muscle rather than increased activity of the triceps muscle (Figure 5A).

#### Stimulus intensity

There was no difference in the level of stimulator output required to evoke the optimal biceps and triceps MEP amplitudes between sides or between groups. Stimulator output was 74.0 ± 4.0% on the more-affected side, 73.5 ± 3.7% on the less-affected side, and 75.5 ± 3.6% in healthy subjects.

#### Stimulus–response curve

**Figure 4:**
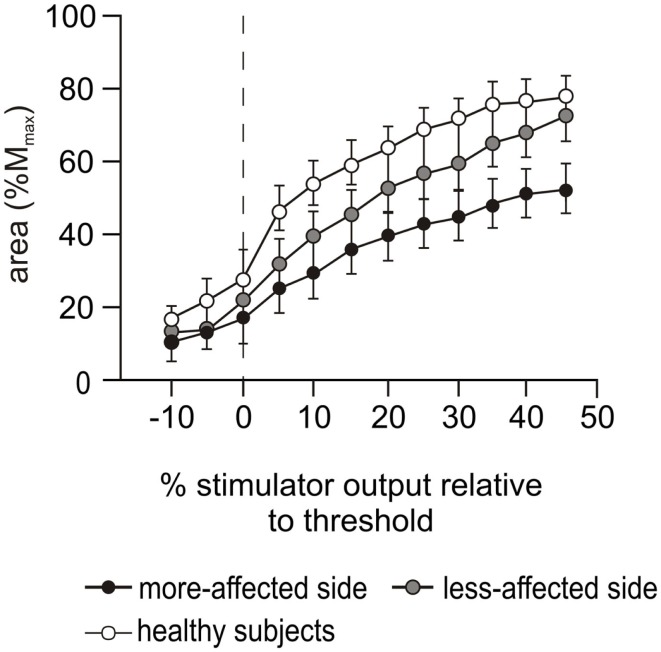
**Stimulus–response curves during cortical stimulation**. Motor evoked potential (MEP) stimulus–response curves during cortical stimulation at 50% MVC for the more-affected (black circles) and less-affected (gray circles) sides of the stroke patients and the combined dominant and non-dominant sides (white circles) of the healthy subjects. Data are presented as mean ± SEM. MEP areas are normalized to the area of M_max_ evoked during Erb’s point stimulation and presented in 5% increments of maximal stimulator output relative to motor threshold. The dashed vertical line indicates motor threshold.

There was an effect for side-tested during the biceps MEP stimulus–response curve collected during contractions of 50% MVC (*p* < 0.001, Figure 4), but there was no interaction between side-tested and stimulus intensity. The curve was sigmoidal for both the more-affected side and healthy subjects, although the MEPs for the more-affected side were smaller at all stimulus intensities above threshold. In contrast, MEPs on the less-affected side continued to increase to the highest stimulus intensity, and were smaller than healthy subjects until 45% stimulator output above threshold when the amplitude for the less-affected side matched that of healthy subjects.

#### Motor evoked potentials during voluntary activation testing

**Figure 5:**
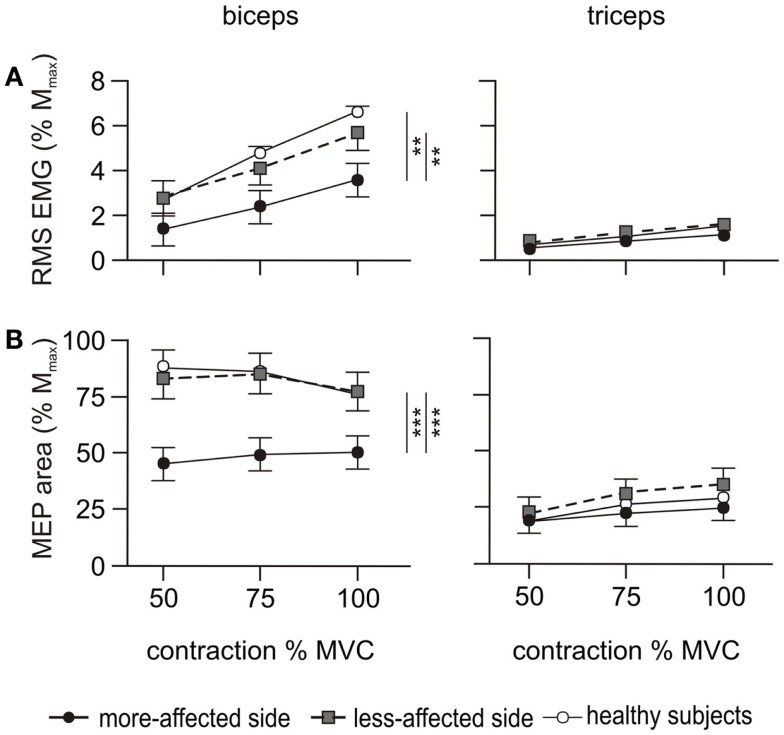
**EMG and MEP during the cortical stimulation protocol**. RMS EMG and MEP area were measured during voluntary contractions of 50, 75, and 100% MVC. Filled circles and solid line represent the more-affected side, shaded circles and dashed line the less-affected side, and open circles and solid line the healthy subjects. The left hand column shows the results for the biceps, and the right hand column the triceps. Data are presented as mean ± SEM. **(A)** RMS EMG over 500 ms prior to the cortical stimulus and normalized to M_max_. The triceps error bars and the data for healthy subjects are obscured by other symbols. **(B)** Area of the MEP evoked by the cortical stimulus normalized to M_max_. The data for each side of healthy subjects are combined. **p < 0.01, ***p < 0.001.

A main effect for side-tested was demonstrated for biceps MEP (*p* < 0.001), but the triceps MEP did not differ (Figure 5B). Biceps MEPs were smaller on the more-affected side compared to both the less-affected side (*p* < 0.001) and healthy subjects (*p* < 0.001), across all contraction levels (50, 75, and 100% MVC). There was no difference in the area between the less-affected side and healthy subjects (Figure 5B). Qualitatively, the biceps MEP area on the more-affected side increased slightly with the contraction level, with the largest MEP occurring at 100% MVC. In contrast, the MEP area on the less-affected side was similar to that of healthy subjects in that the smallest MEPs occurred at 100% MVC (Figure 5B). However, there was no interaction between side-tested and contraction level.

The target amplitude for biceps and triceps MEPs ( >60% M_max_ and <10–15% M_max_, respectively) was only achieved by a single patient on the more-affected side. This increased to 4/10 on the less-affected side. The triceps to biceps MEP ratio demonstrated an effect for side-tested (*p* = 0.019) but no interaction with contraction level. The ratio was smaller on the more-affected side (*p* = 0.015) compared to healthy subjects but was not different between the less-affected side and healthy subjects. Averaged across the three contraction levels the mean triceps to biceps MEP ratio was 52.8 ± 6% on the more-affected side, 41.3 ± 6% on the less-affected side, and 30.3 ± 5% in healthy subjects.

#### Onset latencies

The biceps MEP latency had an effect for side-tested (*p* < 0.001, Table 2). The latency was longer on the more-affected side compared to both the less-affected side (*p* = 0.001) and healthy subjects (*p* < 0.001), with no difference between the less-affected side and healthy subjects. There was no interaction with contraction torque for any side. The onset latency of the triceps MEPs also demonstrated an effect for side-tested (*p* < 0.001) being longer on both the more-affected (*p* < 0.001) and less-affected sides (*p* = 0.024) compared to healthy subjects, and longer on the more-affected side compared to the less-affected side (*p* = 0.018).

#### Silent period

The duration of the silent period evoked during cortical stimulation was measured only in the biceps due to the low level of triceps activity (Table 2). There was an effect for side-tested (*p* < 0.001) with the silent period shorter on the less-affected side compared to both the more-affected side (*p* < 0.001) and healthy subjects (*p* = 0.009). There was no interaction with contraction level, and no difference between the more-affected side and healthy subjects. There was no relationship between the duration of the silent period and the size of the MEP.

*Superimposed twitches* were evoked in all participants using the cortical stimulation protocol. There was an effect for stimulation type (i.e., peripheral nerve versus cortical stimulation) for both the superimposed twitch reported in Newton meter (*p* < 0.001) and when expressed as % MVC (*p* = 0.004). As expected, the amplitude of the superimposed twitch was larger when evoked with cortical stimulation than with peripheral nerve stimulation for both patients and healthy subjects (Figure 3C).

The median amplitude of the superimposed twitch evoked at 100% MVC with cortical stimulation was different between sides-tested when reported in both Newton meter (*p* = 0.012) and when expressed as % MVC (*p* = 0.009) (Figure 3C; Table 2). In both instances, the amplitude was larger on the more-affected side compared to healthy subjects (*p* < 0.05). But there was no difference between sides in the patient group, or between the less-affected side and healthy subjects. There was a non-significant trend for side-tested for the standard deviation of the superimposed twitch amplitude (*p* = 0.07). The more-affected side was significantly larger compared to healthy subjects (*p* < 0.05, Figure 3C; Table 2), but not to the less-affected side. The time-to-peak of the superimposed twitch at 100% MVC demonstrated an effect for side-tested (*p* = 0.032). Time-to-peak was longer on the more-affected side compared to healthy subjects (*p* < 0.05), but not different to the less-affected side (Table 2).

#### Estimated resting twitch and voluntary activation measured with cortical stimulation

**Figure 6:**
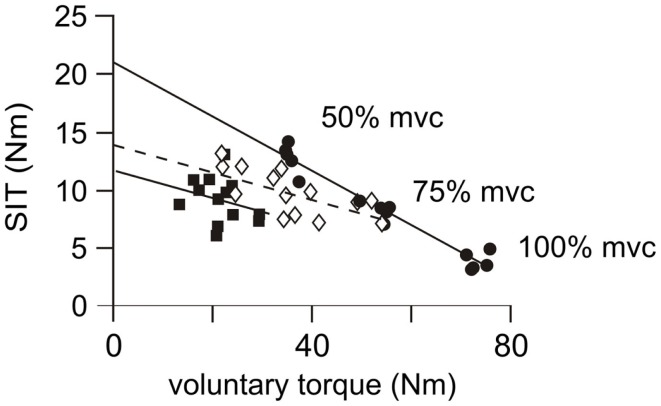
**Individual linear regressions from cortical stimulation**. Examples of linear regressions for the more-affected side of three patients. Subject 1 (filled circles, solid regression line), displays a good linear relationship (*r*^2^ = 0.95, *p* < 0.001) between the magnitude of the voluntary contraction and the amplitude of the superimposed twitch (SIT). Twitch amplitudes are similar in size and appropriately scaled to amplitude of the contraction. This response is similar to those achieved in control subjects and a resting twitch can be reliably estimated from this regression. Subject 2 (open diamonds and dashed regression line) has a moderate linear relationship (*r*^2^ = 0.45, *p* = 0.006). Twitch amplitudes are less-consistently scaled to the strength of the contraction. This regression is unlikely to provide an accurate estimation of the resting twitch. Subject 3 (filled squares and solid regression line), has a poor linear regression (*r*^2^ = 0.08, *p* = 0.350). There is no relationship between the amplitude of the superimposed twitch and the strength of the voluntary contraction so that a resting twitch could not be estimated.

In contrast to healthy subjects, the amplitude of the superimposed twitch for the patient group was not consistently graded to the amplitude of the contraction and there was notable inter-trial variation (Figure 3C). Consequently, the regression coefficients (*r*^2^) between the superimposed twitch and the voluntary torque were often non-linear and ranged from 0.08 to 0.95 (mean 0.55 ± 0.09) on the more-affected side (Figure 6), with only 5/10 being ≥0.80. On the less-affected side regression coefficients ranged from 0.32 to 0.95 (mean 0.72 ± 0.06) with 5/10 being ≥0.80. Thus the estimated resting twitch could not reliably be estimated for either side for patients, and therefore the cortical voluntary activation score could not be calculated. In contrast, regression coefficients for the healthy subjects were between 0.80 and 0.97 (mean 0.91 ± 0.26). The mean estimated resting twitch amplitude was 19.0 ± 1.1 Nm, with a median voluntary activation score of 91.3% (85.6–96.1%).

## Discussion

This study is the first to use both peripheral and cortical stimulation techniques to assess voluntary activation in the elbow-flexor muscles on both sides of stroke patients and healthy age-appropriate control subjects. The contractile properties and EMG characteristics of the stimulated muscles were also examined to suggest where in the motor pathway post-stroke impairments occurred. Although impairments in the voluntary activation of the less-affected side have been demonstrated previously in the lower limb post-stroke, the results of this study suggest the bilateral impairments in voluntary activation in the upper limb are the result of distinct differences in neural drive to the more- and less-affected side. Peripheral nerve stimulation revealed significant reductions in voluntary activation of the elbow flexors on both the more- and less-affected sides after stroke compared to healthy age-matched subjects. However, no differences in the contractile properties were evident from twitches (in Newton meter) evoked at rest by this stimulation, suggesting reduced neural drive to the muscle is a greater contributor to post-stroke impairments in this cohort than changes to the muscle itself. Cortical stimulation was completed in all patients, although the inconsistency of the superimposed twitch (Figure 6) meant voluntary activation scores could not be calculated with certainty using this method. Regardless, cortical stimulation was the only means of revealing the important, but distinct differences in the connectivity of the corticospinal network of *both* the more- and less-affected sides of stroke patients during a MVC. Descending pathways on the less-affected side could be activated by stimulation, but were not being fully driven voluntarily. In contrast, cortical stimulation of the more-affected cortex suggested impairments both to the descending corticospinal connections, and to the ability to voluntarily drive muscles through those connections.

### Methodological considerations

This is the first study to implement both peripheral nerve and cortical stimulation voluntary activation protocols in the same way for both patients and healthy control subjects. The methods and muscles studied were chosen to replicate as closely as possible the work of Todd and colleagues (19, 20), who first implemented and validated the cortical stimulation protocols in healthy subjects. The single stimulus used for peripheral nerve stimulation may have affected the measured level of voluntary activation, but this influence was probably minimal given the high MVC levels performed in this study (17). In addition, the elbow flexors can be readily stimulated by both methods, are less influenced by hand dominance than the more distal muscles due to greater bilateral projections (44), and can be activated even by stroke patients with profound weakness. The methods were modified slightly from those used in younger subjects (20) to minimize fatigue and discomfort for the stroke patients. Firstly, the number of maximal contractions was limited to 5 rather than the usual 10. Patients also had more inconsistent maximal torque generation on the more-affected side, and often took longer to reach the target level although care was taken to ensure the torque plateaued before the stimulus was given. Previous studies have reported a decrease in the ability to maintain force steadiness in the upper-limb after stroke (45), but there was no significant difference between the force steadiness of the relatively well-recovered patients and healthy subjects in this study. Despite the variability of MVCs, patients were able to appropriately grade submaximal efforts and there was no difference in the mean torque generated at 100% MVC during the two stimulation protocols. This suggests that fatigue did not contribute to the differences in muscle properties measured between sides or groups.

The necessary methodological differences that occurred during the cortical stimulation protocol between patients and healthy subjects may also have influenced our results. When stimulating over the vertex during a 50% MVC, the desired biceps and triceps MEP target amplitudes ( >60% M_max_ and <15% M_max_, respectively) were not achievable in most patients, despite selection of the stimulation site over the cortex generating the largest biceps and smallest triceps MEPs. Similarly, we may have *underestimated* the extent of voluntary activation decline due to the relatively high-functioning status of the stroke cohort (Table 1). Although we endeavored to recruit patients with low-functional ability, they were unable to participate due to TMS contraindications or an inability to be positioned in the myograph.

Due to the characteristics of the small cohort examined in this study, our results can only be generalized to well-recovered patients after stroke. Although the use of the more-affected upper arm was quantified by the MALQOM as moderate to high (Table 1), we did not record general activity levels, which may have contributed to our findings. We assumed that the stroke patients were neurologically healthy prior to their stroke, but it is feasible they had pre-morbid impairments in vascular or neuromuscular function. Further, 6 of the 10 stroke patients were on statin medications, which may have affected their muscle strength, but presumably not their level of voluntary activation. It is not known if any of the healthy subjects were on similar medication. Finally, we acknowledge our findings may be related to the location and size of the stroke lesions but this could not be investigated as this information was not available for all patients.

### Physiological changes to muscle after stroke

Maximal voluntary torque was significantly reduced on both the more- and less-affected sides of stroke patients compared to healthy subjects. Maximal Mwave amplitudes were also reduced in the stroke patients and this suggests a deficit in the muscles. However, this was not apparent in evoked force responses. Surprisingly, there were no differences in the contractile properties of the biceps resting twitch between groups, despite considerable variation in the amplitude of the five twitches in some patients (Figure 3A). To our knowledge, a similarity in resting twitch properties between healthy subjects and stroke patients has not been reported previously in the upper-limb. Conflicting results describing resting twitch amplitudes have however been reported in the lower limb (26, 46). This may reflect a shift to a higher proportion of either type I (47) or type II muscle fibers (48) post-stroke. We also saw no overt evidence of muscle atrophy as only two patients had a difference in arm circumference >5% (6 and 9%, Table 1). We recognize that this is only a crude estimation and that a bilateral similarity does not necessarily equate to maintenance of lean muscle mass. It is feasible that increases in non-contractile tissue such as fat and connective tissue had occurred (49, 50), but if this were the case it did not appear to affect the contractile properties of the stimulated muscle. These findings suggest that changes in the properties of the elbow-flexor muscles were not a significant contributor to the decreased torque levels in this study (23, 51, 52), but reductions in voluntary drive were of greater significance.

Despite the similarities in the contractile properties of the muscle, an increase in muscle resistance or passive stiffness as measured with the modified Ashworth scale (Table 1) was apparent in eight stroke patients. Five patients had difficulty relaxing their more-affected biceps immediately after contractions and three patients had increased single motor unit activity evident in surface EMG recordings from the more-affected side throughout testing. Although care was taken to ensure that force had returned to baseline levels before the resting twitch was evoked, hypertonicity at rest (modified Ashworth scores, Table 1), may have contributed to resting twitch amplitudes that were similar to those of healthy subjects and may have contributed to the considerable within-subject variability in the resting twitch. Increased muscle resistance may have resulted in an *overestimation* of the voluntary activation score on the more-affected side of some patients. Regardless our results still demonstrate significant impairments in this relatively high-functioning cohort. An increase in muscle resistance in the more-affected biceps may also help to maintain lean muscle mass despite reduced descending drive. An increase in spasticity has been linked to greater muscle mass after stroke (50), although there is little other evidence supporting this hypothesis at present.

### Voluntary activation measured with peripheral nerve stimulation after stroke

Voluntary activation scores calculated from peripheral nerve stimulation were significantly reduced on both the more- and less-affected sides of stroke patients compared to healthy subjects. Bilateral voluntary activation deficits have been reported previously in leg muscles after stroke (22, 23), but not in arm muscles, which have finer neural control properties. The only previous study undertaken in the elbow flexors (27) did not include healthy age-matched subjects from which to make a comparison. It is unlikely that the changes in voluntary activation after stroke are due to age as only minimal age-related changes have been reported in older subjects previously (30–35). Stroke-related impairment of voluntary activation independent of age is further supported by the results reported here.

The reduced voluntary activation identified through peripheral nerve stimulation indicates that the motoneurones, and therefore the muscle, are not being driven adequately on either the more- or less-affected side post-stroke. This may be due to reductions in the number of neural connections, or it could be that the connections are intact but are not being fully driven (16–18).

### Voluntary EMG

The reduced M_max_ amplitude on both sides post-stroke suggests there may be some peripheral deficit, but the use of peripheral nerve or Erb’s point stimulation alone cannot distinguish between changes in the functional connectivity and anatomy. Thus voluntary EMG and muscle responses to cortical stimulation were examined in detail to investigate these mechanisms after stroke and in healthy controls. EMG was reduced post-stroke on the more-affected but not the less-affected side compared to healthy subjects. The reduction in voluntary activation on the more-affected side was consistent with decreased EMG activity compared to both the less-affected side and healthy subjects. This reduction in voluntary EMG presumably reflects not only reduced descending drive but also post-stroke impairments in the firing rate, rate modulation, and recruitment of motoneurones (53–55). On the less-affected side, the voluntary EMG appears similar to healthy subjects when the EMG is normalized to the size of the maximal M wave. However, the reduction in voluntary activation suggests that changes in the firing and recruitment properties of the motoneurones may not be detected by recordings through surface electrodes. The EMG findings also suggest that the change in elbow-flexor activity on both the more- and less-affected sides was a more important contributor to the apparent increase in co-contraction than an increase in the activity of the elbow extensors. Reduced agonist activation contributes more to reduced force output post-stroke than increased antagonist activation (23, 51, 52).

### Torque and EMG responses to cortical stimulation after stroke

Superimposed twitches were evoked for all participants using cortical stimulation (Figure 3C) but the inconsistent relationship between voluntary torque and the size of the superimposed twitch meant that voluntary activation could not be calculated with certainty for the stroke group using this protocol. The larger superimposed twitch on the more-affected side for the pooled data imply that voluntary cortical output was not sufficient to drive the motoneurones (and the muscle) and also that some cortical capacity remained untapped by voluntary effort but could be activated by TMS. The small superimposed twitches seen in individual patients (Figure 3C) may reflect a methodological problem with activation of both the elbow extensor and flexor muscles by the cortical stimulus. Alternatively, it is probable that some patients have good voluntary recruitment of surviving descending connections that leaves little cortical capacity untapped, despite insufficient corticospinal neurones to enable full recruitment of the motoneurones and muscle. The variability of within-patient cortically evoked superimposed twitches (Figure 3C) that were seen on both sides after stroke may reflect changes in the cortex and spinal cord. It is not possible to know how the stroke lesion itself, or the plastic changes to neural connections (56, 57), may affect distribution of the TMS stimulus within the cortex. It is possible that different neural connections are activated during each stimulus resulting in inconsistent excitation of corticospinal pathways and variable twitch amplitudes (58).

We examined MEPs to ascertain possible differences in the connectivity of the descending corticospinal tract. The latencies of the MEPs on the more-affected side were prolonged and the area of the maximal MEP on the more-affected side was reduced compared to the less-affected side and healthy subjects. Differences were apparent during both the cortical stimulus–response curve at 50% MVC (Figure 4) and the maximal contractions performed during the voluntary activation trials (Figure 5B). Smaller, delayed MEPs on the more-affected side in the chronic period of stroke are consistent with previous studies (58). Qualitatively, the largest MEP was seen at 100% MVC (Figure 5B) rather than the 50 or 75% MVC seen in the healthy subjects in this study, and reported previously (19, 20). From this study, it was not possible to determine exactly why, or where in the motor pathway impairments contributing to this pattern occurred. We assume that the reduced MEPs on the more-affected side are primarily due to the loss of cortical neurones post-stroke, although diaschisis can also reduce motor cortex excitability (10, 11). Diaschisis is the disruption in brain function of areas remote from the lesion caused by the withdrawal of neural inputs from damaged areas (59, 60). Although the initial stroke lesion does not damage peripheral pathways directly, changes in connectivity within the remaining cortex can arise from factors such as collateral sprouting, re-routing of neural pathways, and changes to the excitability of neuronal circuits [see in Ref. (61)]. Long-term reductions in descending drive and changes to spinal reflexes (62–64) may impair the functional connectivity of descending tracts (12) or induce plastic changes at the level of the motoneurone (55, 65, 66).

Similar to the voluntary EMG, the area of the biceps MEPs on the less-affected side of patients was not different to that of healthy subjects (Figure 5B), nor was its latency prolonged. This suggests that the less-affected corticospinal tract could be stimulated with the same efficacy as in healthy subjects. However, in the cortical stimulus–response curve at 50% MVC (Figure 4), a marked difference in the MEP response was seen between the healthy and patient groups. The less-affected MEP was smaller than healthy subjects at lower stimulation intensities, but reached the same area at the highest intensity. In healthy subjects, the stimulus–response curve reached a plateau at 35% of stimulator output above threshold (Figure 4). The MEP area depends on the integrity of the corticospinal fibers, the strength and number of synapses to the motoneurones, and the ability of the TMS to evoke repetitive activity whereas the plateau represents the maximal corticospinal output available to the TMS stimulus (1). The reasons for the stimulus–response curve pattern on the less-affected side are unclear as we assume there was no direct damage to the contralesional cortex of these patients who had suffered a unilateral stroke. The size of MEPs was similar when contraction strengths and/or stimulus intensities were high, and this suggests a functional rather than structural impairment.

## Conclusion

This is the first study to demonstrate reductions in upper-limb voluntary activation and maximal torque on both the more- and less-affected sides in patients with relatively high motor-function post-stroke, compared to healthy age- and sex-matched control subjects. Comparisons between the results of the peripheral and cortical stimulation methods suggest the bilateral voluntary activation impairments measured are caused by distinct impairments in neural drive. Peripheral nerve stimulation did not reveal differences in the contractile properties of the elbow-flexor muscles at rest. In contrast, the voluntary activation score could not be calculated with certainty with cortical stimulation due to the inconsistency of the superimposed twitches. Irrespective, examination of the EMG results from the cortical stimulation showed significant bilateral, yet distinct differences in the neural drive after stroke, and in the connectivity of the descending motor pathway on the more-affected side. We suggest that the expected muscle weakness on the more-affected side of stroke patients was due to impairments of the descending corticospinal connections, coupled with an inability to drive through those connections. In contrast, descending connections to the less-affected side appeared to be intact, but the weakness was due to an inability to voluntarily drive the connections to the muscle. This study suggests quantification of voluntary activation with cortical stimulation is not possible in patients after stroke. Despite this finding, cortical stimulation revealed changes in the neural drive and descending tracts in this cohort with high motor-function that could not be measured when using peripheral nerve stimulation alone. These findings are presumably substantially greater in patients with less motor-function. The impairments in cortical networks and descending pathways identified in this study highlight the importance of neurorehabilitation strategies that target both sides of the body.

## Conflict of Interest Statement

The authors declare that the research was conducted in the absence of any commercial or financial relationships that could be construed as a potential conflict of interest.
